# On tests of activation map dimensionality for fMRI-based studies of learning

**DOI:** 10.3389/fnins.2015.00085

**Published:** 2015-04-14

**Authors:** Juemin Yang, Lior Shmuelof, Luo Xiao, John W. Krakauer, Brian Caffo

**Affiliations:** ^1^Department of Biostatistics, Bloomberg School of Public Health, Johns Hopkins UniversityBaltimore, MD, USA; ^2^Department of Brain and Cognitive Sciences, Ben-Gurion University of the NegevBeersheba, Israel; ^3^Departments of Neurology and Neuroscience, Johns Hopkins UniversityBaltimore, MD, USA

**Keywords:** canonical variates analysis, cognitive learning, BOLD fMRI, statistical parametric mapping, interaction test

## Abstract

A methodology for investigating learning is developed using activation distributions, as opposed to standard voxel-level interaction tests. The approach uses tests of dimensionality to consider the ensemble of paired changes in voxel activation. The developed method allows for the investigation of non-focal and non-localized changes due to learning. In exchange for increased power to detect learning-based changes, this procedure sacrifices the localization information gained via voxel-level interaction testing. The test is demonstrated on an arc-pointing motor task for the study of motor learning, which served as the motivation for this methodological development. The proposed framework considers activation distribution, while the specific proposed test investigates linear tests of dimensionality. This paper includes: the development of the framework, a large scale simulation study, and the subsequent application to a study of motor learning in healthy adults. While the performance of the method was excellent when model assumptions held, complications arose in instances of massive numbers of null voxels or varying angles of principal dimension across subjects. Further analysis found that careful masking addressed the former concern, while an angle correction successfully resolved the latter. The simulation results demonstrated that the study of linear dimensionality is able to capture learning effects. The motivating data set used to illustrate the method evaluates two similar arc-pointing tasks, each over two sessions, with training on only one of the tasks in between sessions. The results suggests different activation distribution dimensionality when considering the trained and untrained tasks separately. Specifically, the untrained task evidences greater activation distribution dimensionality than the trained task. However, the direct comparison between the two tasks did not yield a significant result. The nature of the indication for greater dimensionality in the untrained task is explored and found to be non-linear variation in the data.

## 1. Introduction

This manuscript considers settings where task-related activation may be present before and after learning, yet the distribution of activated voxels changes. For context, consider the motivating study for the work, where two motor tasks of equal difficulty were performed in a scanner over two sessions. Training for one of the tasks occurred in between the sessions, while the other task served as a control. Current methodology would use random effects statistical parametric mapping (SPM Friston et al., 2011) to test for a differential effect of training between tasks to study learning. However, this approach suffers from considering only voxel-level activation, or change in activation, in isolation. In contrast, learning may induce changes in *activation distribution*, i.e., the distribution of intensities of BOLD responses to the paradigm. Moreover, the study of activation distributions offers many potential benefits over voxel-level testing, including: the elimination of multiplicity concerns, robustness to registration, and sensitivity to hypotheses of particular interest in the study of learning.

Analysis of dimensionality of fMRI task-based activation maps (Worsley et al., 1997; Zarahn, 2002) provides a starting framework. The proposed procedure considers the distribution of activation maps and tests their dimensionality using eigenvalue decompositions. To illustrate the goals of the test, consider our motivating example. Learning could manifest itself in many ways in the collection of voxels that are activated. For example, BOLD contrast estimates of the activated voxels could be identical across sessions, increased or decreased, change from activated to not (and vice versa) or uncorrelated. The test of dimensionality should be considered one of several possible probes to interrogate such hypotheses.

Our investigation includes a large scale simulation study of brain activation maps. The simulation results demonstrate that the study of dimensionality in a framework similar to Zarahn (2002) is able to capture learning effects. The motivating data set is used to illustrate the method, which is applied to the trained and untrained tasks separately and then jointly.

## 2. Methods

Subjects performed an fMRI motor task in two scanning sessions, with training between them. A second, similarly difficult, fMRI motor task was performed at the two sessions, but had no training in between. We focus on activation maps within an appropriately selected spatial mask, such as one encapsulating the primary motor cortex. Let $\hat{\gamma }$_*ijk*_(*v*) be the subject- (represented by index *i* = 1, …, *N*), session- (*j* = 1, 2), task- (*k* = 1, 2) and voxel- (*v* = 1, …, *V*) specific estimates of task activation. These are obtained by voxel-wise regression of a HRF-convolved task paradigm in registered space (see Lindquist, 2008; Lindquist et al., 2009, for descriptions and discussion), conducted separately for each subject's visit.

This paper is concerned with the statistical analysis of, and hypotheses associated with, the collection of subject-specific activation maps, represented by the *V* × 2 matrix ${\hat{\Gamma }}_{ik}={\left\{{\hat{\gamma }}_{i1k}(v),{\hat{\gamma }}_{i2k}(v)\right\}}_{v=1}^{V}$.

A conceptual model is considered where the activation maps are estimates of assumed true activation maps, Γ_*ik*_ = {γ_*i*1*k*_(*v*), γ_*i*2*k*_(*v*)}^*V*^_*v* = 1_. Thus, variation in the elements of Γ_*ik*_ is (intra-subject) biological variation in the hemodynamic BOLD response to the paradigm. In contrast, variation in $\hat{\Gamma }$_*ik*_ includes this biological variation, as well as all of the variation and biases that occur in the practical process of computing the BOLD paradigm response.

Both $\hat{\Gamma }$_*ik*_ and Γ_*ik*_ also vary across subjects. Consider the *V* × 2 matrix, *A*_*k*_ = {β_1*k*_(*v*), β_2*k*_(*v*)}^*V*^_*v* = 1_ as representing the population average of voxel-level activation. Here β_*jk*_(*v*) = E(γ_*ijk*_(*v*)), *j* = 1, 2. A non-zero β_*jk*_(*v*) indicates that, on average, subjects activated at that particular location. Treating *v* as being meaningfully consistent across subjects requires that appropriate template-based (or equivalent) registration has been performed. The matrix, $\hat{A}$_*K*_, is thus a data-level estimate of *A*_*k*_, obtained by taking empirical means across subjects at each voxel.

A straightforward investigation of learning for the first (trained) task arises from a sharp null hypothesis test of:

$$H_{0}:\beta_{21}(v)-\beta_{11}(v)=\beta_{22}(v)-\beta_{12}(v),$$

conducted separately, voxel-by-voxel. This tests the difference in the longitudinal change in the BOLD response between the trained and untrained tasks. Comparing longitudinal learning effects with a reference (untrained) task addresses non-learning based biases across sessions. The test in question is normally conducted with standard interaction tests—perhaps accounting for subject-level correlation (see Diggle et al., 2002, for a general treatment of correlated data). Typically, the test is performed separately at each voxel, via so-called Statistical Parametric Mapping (SPM). Significance is usually ascertained with super-threshold voxel level statistics using random field theory (see Friston et al., 2011, and the references therein) or via resampling statistics (Nichols and Holmes, 2001).

This SPM approach has several benefits for the study of learning. However, it also has limitations. Notably, the approach suffers from multiplicity issues and concentrates only on focal and localized interaction hypotheses, one voxel at a time. Moreover, it is highly dependent on accurate co-registration across subjects. Little information is gained from the ensemble of voxels, except through smoothing during preprocessing.

As an alternative, consider examining the activation distribution. Let *D* = *A*_2_ − *A*_1_ = {β_21_(*v*) − β_11_(*v*), β_22_(*v*) − β_12_(*v*)} be the *V* × 2 matrix of longitudinal changes in the contrasts of interest, with its associated estimate, $\hat{D}$. The SPM approach tests whether the two entries of each row of *D* are the same. Suppose one instead assumes that elements of *D* arise from a bivariate distribution and interest is in the ensemble of voxel-specific pairs, instead of individual voxels.

**Figure 1:**
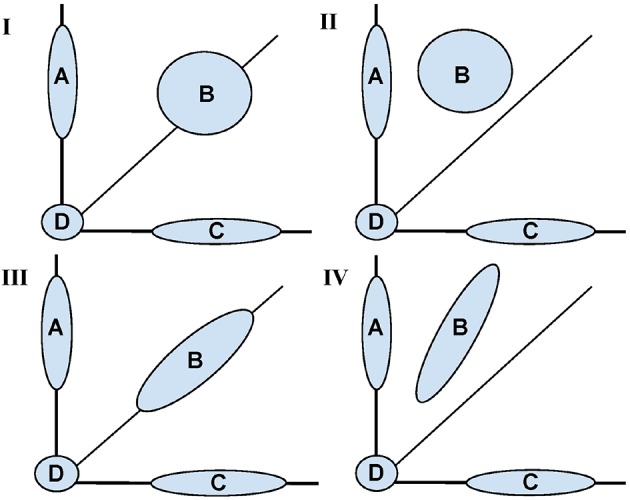
**Conceptual diagram for fMRI activation distributions based on the motivating study of motor learning**. Shaded areas represent learning based (inter-session differences) between a trained (Y axis) and untrained (X axis) task. Across all panels, Area (A) represents voxels with change in activation across sessions only in the trained task, (B) represents voxels with change in activation across sessions in both the trained and untrained task, (C) voxels with change in activation across sessions only in the untrained task, (D) represents no change in activation for both tasks. The four panels **(I–IV)** represent different potential shapes of the activation distributions for (B) with **(I, II)** showing a two dimensional shape and **(III, IV)** showing an approximately one dimensional. In **(I, III)** inter-sessions differences are symmetrically represented whereas in **II, IV** one task had a uniformly greater increase.

Figure 1 is a conceptual diagram showing possible shapes associated with the distribution of voxel pairs. The conceptual model is informed by the idea of Gaussian mixture models (see McLachlan and Peel, 2000, for an introduction). The mixture model is governed by four major areas: (A) voxels that were “activated” (had a change across sessions) only in the trained task, (B) voxels that were activated in both tasks, (C) voxels that were activated only in the untrained task, and (D) voxels that were not activated in either task.

It is the shape of (B) that is of primary interest. For instance, any shift in B above the diagonal line represents training based learning. If the shape is spherical, there is no correlation between training status and change in activation across sessions. In contrast, the more ellipsoidal the shape, the greater the correlations in activation extent across sessions.

While acknowledging that SPM operates voxel-by-voxel, and that Figure 1 displays voxel groups, the SPM approach would investigate each point's distance from the diagonal line, assessing significance relative to inter-subject variability. Therefore, given enough data, the SPM approach would conceptually reject for voxels in groups (A) and (C) in the cases represented by all panels. However, it would reject most of the voxels in group B in panels II and IV only. The approach would reject few of the voxels in (B) for panels I and III. Contrast this with the shape and dimensionality of (B) being constant for panels I and II together and III and IV together. Thus, to the extent that learning represents itself as changes in the shape of the activation distribution, the voxel-wise approach would not tell the complete story.

Instead, we view the shape of the bivariate distributions of points in group (B) as informative for studying changes in task activation. One key attribute is its intrinsic dimensionality (1 vs. 2 dimensional). Ignoring groups (A), (C), and (D), one would conclude that (B) is two dimensional in panels I and II and intrinsically one dimensional in III and IV. The dimensionality of (B) is useful for differentiating whether changes in intensity or distribution account for activation changes following learning.

The use of principal components to investigate the dimensionality of learning builds upon an existing literature on the use of dimensionality testing in the study of activation maps (Worsley et al., 1997). Specifically, Zarahn (2002) and Moeller and Habeck (2006) considered it within the context of functional imaging. The aim of this work is to study the goals, limitations and hypotheses of tests of dimensionality of fMRI activation maps. A test of one vs. two dimensions on the set $\hat{D}$, that is rank($\hat{D}$), investigates the null hypothesis

$$H_{0}:\beta_{21}(v)-\beta_{11}(v)=c\{\beta_{22}(v)-\beta_{12}(v)\}$$

for unspecified *c* and collectively for all voxels *v*.

Let ${\hat{A}}_{k}=\frac{1}{N}\sum {\hat{A}}_{ik}$ and recall that $\hat{D}={\hat{A}}_{2}-{\hat{A}}_{1}$. Following the existing work on tests of dimensionality in fMRI, we use root tests of the second eigenvalue (see Mardia et al., 1980) to investigate the hypotheses of one dimension vs. two. A simulation-based investigation of this test follows. The simulation study includes: the strength of the effect, the intrinsic dimensionality (considering power and error rates), and the impact of biological and measurement variation, including variation in the angle of the subject-specific principal direction.

## 3. Materials and simulation

### 3.1. Motivating data set

**Figure 2:**
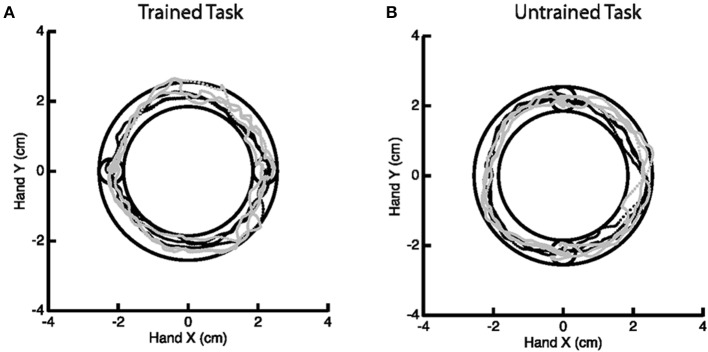
**Example of the Arc Pointing Task (APT) executed within the fMRI session**. Subjects were asked to navigate a cursor lying between the inner and outer concentric circles. Two tasks of similar difficulty were investigated. A horizontal task **(A)** where subjects were trained in between two scanning sessions and a vertical task **(B)** where subjects were not trained.

A motor learning study served as motivation for this work, though we emphasize that the methodology generally applies to any study of change in activation. The goal of the motor study centered on investigating skilled motor learning via the Arc Pointing Task (APT) (Shmuelof et al., 2012), where the task was designed to better understand neural correlates of motor skill acquisition. The subjects completed two similarly demanding motor tasks of drawing an arc within reference lines by moving their (non-dominant in all cases) left wrist. The interior circles in Figure 2 represent the starting and end points of the path. Subjects were directed to stay within the lines of the outer circles while tracing the arc. Subjects were scanned while performing the tasks at baseline and again 5 days later, with training on just one of the two tasks in the interim. Comparison of fMRI activation (or any measurement of motor function) from baseline to follow-up considers both effects related to motor learning and those related to changes between sessions. Comparison with the, otherwise similar, untrained task as a reference eliminates additive inter-session biases unrelated to learning.

The specifics of the study are as follows. Thirteen right-handed subjects (8 females, 18–27 years of age) engaged in the above described motor tasks, none having performed these tasks previously. Subjects participated in a 5 day protocol consisting of daily behavioral sessions in the lab and two fMRI scans on the baseline and final days (1 and 5, respectively). During scanning, subjects performed the APT. Horizontal (trained) and vertical (untrained, control) APT movements were performed in separate block design experiments before and after training for the horizontal task. Six movements were performed in 18 blocks (repeated 6 times), at a slow speed (1.5 s per movement). Subjects received online feedback regarding the position of the cursor, but no further information about their success or failure, or about their movement speed. In the trained task, targets were presented on the horizontal line (same configuration as during the behavioral task in the lab) and in the untrained task, targets were aligned vertically. Movements were always in the clockwise direction. Subjects performed the movements with their (non-dominant) left wrist, while lying on their back, and receiving visual feedback of their movements through goggles (resonance technology, Los Angeles, CA). Further details on the experimental paradigm can be found in Shmuelof et al. (2014).

Data was acquired on a Philips Intera 3T scanner using a Philips SENSE head coil. The functional scans were collected using a gradient echo EPI, with voxel size of 3 × 3 × 3 mm (240 × 240 × 240 mm matrix). *TR* = 2 s, flip angle = 77^*o*^, axial slices, *TE* = 25 ms. *Forty* slices were gathered in an interleaved sequence at a thickness of 3 mm (no gap). *Ninety* − *six* volumes were accumulated in each experimental run. The first 2 volumes were discarded to allow magnetization to reach equilibrium. A single T1-weighted anatomical scan was also obtained for each subject (MPRAGE, 1 mm^3^).

**Figure 3:**
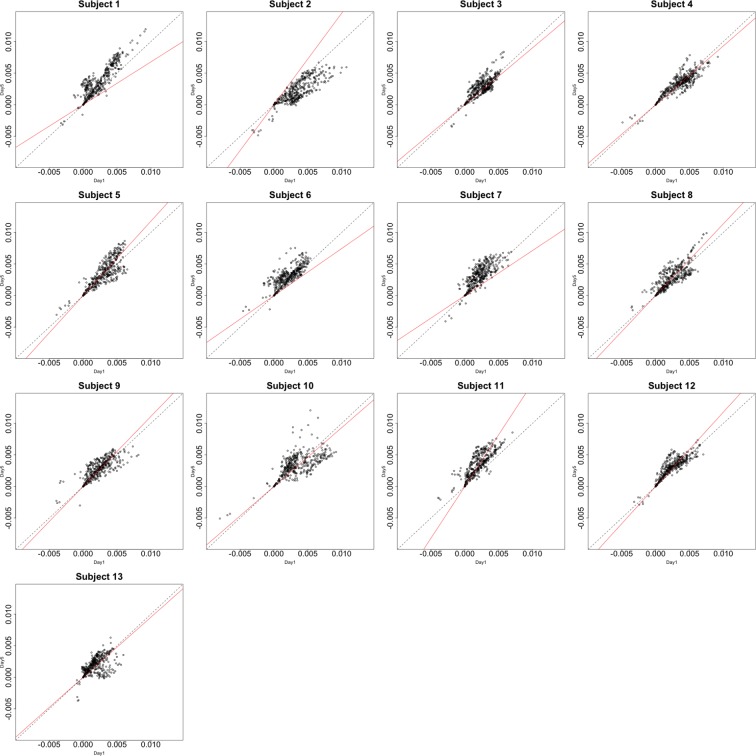
**Contrast maps from the horizontal arc pointing task**. The X axis for each plot is the first session while the Y axis is the second. Red lines show the direction of the first principal component while a dotted identity line is shown for reference.

**Figure 4:**
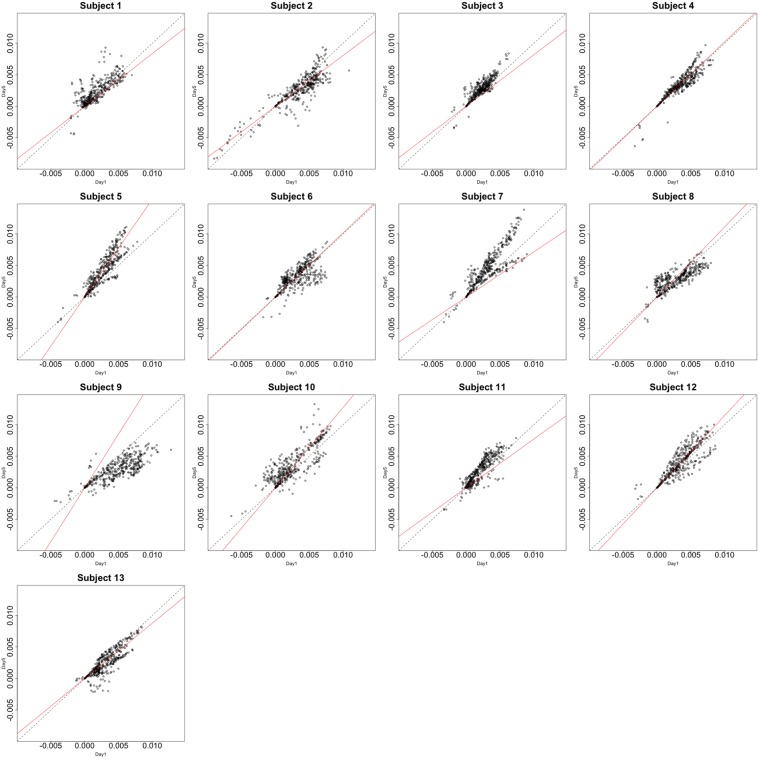
**Contrast maps of the vertical arc pointing task**. The X axis for each plot is the first session while the Y axis is the second. Red lines shows the direction of the first principal while a dotted identity line is shown for reference.

Functional data were preprocessed using SPM5 (http://www.fil.ion.ucl.ac.uk/spm/software/spm5/). Before statistical analysis, the data was also corrected for slice timing acquisition and head motions, re-sliced to 2 × 2 × 2 mm voxels using a fourth degree B-spline interpolation, and transformed into a Talairach standard space (Talairach and Tournoux, 1988). A general linear model was used for data analysis, followed by calculation of beta maps. Scatter plots of beta before training and after training are shown in Figures 3, 4.

By comparing the trained and untrained tasks, the population impact of learning was estimated by considering differences in the change in activation maps over sessions. Using the developed notation, the collections compared are, {β_21_(*v*) − β_11_(*v*)}_*v* = 1, … *V*_ to {β_22_(*v*) − β_12_(*v*)}_*v* = 1, …, *V*_, where, as previously noted, the first index indicates session (baseline and fifth day) and the second indicates task (horizontal and vertical). The test of dimensionality then considers whether the changes in activated voxels after training is uncorrrelated with the changes in the untrained (but otherwise similar) task. Under Gaussian assumptions, absence of correlation among activated voxels implies that the extent of activation is unrelated between sessions.

All subjects gave written, informed consent and received a small compensation for participating in the Study, which was approved by the Columbia University Institutional Review Board.

### 3.2. Simulation study

Assume there are *V* = *V*_1_ + *V*_2_ voxels in total: *V*_1_ that are significantly different across sessions (group B in Figure 1) and referred to as “activated,” and *V*_2_ that are not (group D in Figure 1). Under this working example, the term activated implies a non-zero change in the contrast values across sessions. Thus, $\pi =\frac{V_{2}}{V}$ is the percentage of non-activated voxels.

The simulation model is:

(1) $$b_{iv}\overset{iid}{\sim }N\;\left\{\left(\begin{array}{c} \beta_{21}(v)-\beta_{11}(v)\\ \beta_{22}(v)-\beta_{12}(v) \end{array}\right),I\sigma^{2}\right\}=N(\delta (v),I\sigma ),$$

where δ(*v*) = {δ_1_(*v*), δ_2_(*v*)} = {β_21_(*v*) − β_11_(*v*), β_22_(*v*) − β_12_(*v*)} and *b*_*iv*_ = {*b*_1*iv*_, *b*_2*iv*_} is a subject-specific realization plus noise. The generation of the δ(*v*) parameters varied across simulation settings, and is described separately for each case below.

In all simulation settings, the estimate of the *V* × 2 matrix of the δ(*v*), labeled $\hat{D}$, was obtained via the voxel-specific mean across subjects. Following Worsley et al. (1997), the *V* × 2 matrix, *Z*, denotes $\hat{\delta }$ divided by its standard error. That is, $Z_{k}(v)=\text{Var}{\left\{{\hat{\delta }}_{k}(v)\right\}}^{-1/2}{\hat{\delta }}_{k}(v)$ make up row *v* and column *k* of *Z*. Here the variance was calculated across subjects separately for each voxel. The cross-product matrix is then

$$S=\sum_{v=1}^{V}Z{\left(v\right)}^{\prime }Z(v)/V.$$

The Lawley/Hotelling trace statistic is:

$$S_{q}=\sum_{j=q+1}^{h}\lambda_{j}/(h-q),$$

where λ_*j*_, *j* = 1, 2, …, *h* are the eigenvalues of *S*, *h* is the total number of eigenvectors and *q* is the testing rank. Under independence and Gaussian assumptions, *S*_*q*_ follows an *F* distribution under the null hypothesis, where the first *q* principal components capture all of the signal. In our case, *h* = 2, *q* = 1 and the test statistic is simply the second eigenvalue of *S*.

#### 3.2.1. Simulation under the null hypothesis

The first simulation setting considers the hypothesis of unidimensionality; that is, whether δ_1_(*v*) = *c*δ_2_(*v*), where *c* is constant across subjects. The parameter δ_1_(*v*) for the activated voxels was simulated as uniformly distributed in [*min*, *max*], with this range computed from values of [0, 1]–[10, 15]. Note that for voxels inactive in both time points, δ_1_(*v*) = 0. Thus, δ_1_(1), …, δ_1_(*V*_1_) ≠ 0 while δ_1_(*V*_1_ + 1), …, δ_1_(*V*) = 0. Note that, δ_2_(*v*) = *c*δ_1_(*v*) regardless of null status.

**Figure 5:**
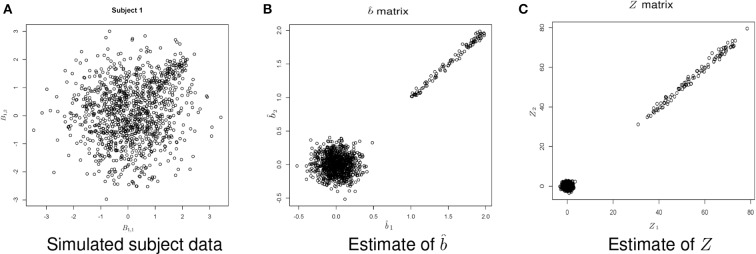
**Example simulated data**. **(A)** Shows the simulated data. **(B)** Shows the estimate of *b* using Equation (1). **(C)** Shows the estimate of *Z*, where $Z_{k}(v)=\text{Var}{\left\{{\hat{\delta }}_{k}(v)\right\}}^{-1/2}{\hat{\delta }}_{k}(v)$.

Figure 5 shows example data for a simulated subject as well as the estimated statistics. The null simulation varied according to the following: (*i*) distance of the activated voxels from the inactivated ones, as well as the range of activation, (controlled by *min* and *max*); (*ii*) the percentage of inactivated voxels (π); and (*iii*) the number of subjects (*N*). For all of the null hypothesis scenarios, *c* = 1. The type I error rates correspond to the percentage of rejections of the Lawley/Hotelling trace statistic for each simulation setting. The specifics of each scenario are described below while the results are shown in Table 1.

**Simulation under variation in the distance:** In this scenario, *N* = 12, *V*_1_ = 40, *V*_2_ = 200, and σ = 1. Five scenarios for each pair of *min* and *max* were considered. The results suggest that the type I error is not significantly affected by the distance of the activated voxels from the inactivated ones.
**Changing the percentage of inactivated voxels:** In this case, *N* = 12, σ = 1, *min* = 0.5, and *max* = 1.5. The total number of voxels was set at *V* = 240. These results suggest that the test is not significantly affected by the percentage of inactivated voxels.
**Varying the number of subjects:** In this case, *V*_1_ = 40, *V*_2_ = 200, σ = 1, and [*min*, *max*] = [0.5, 1.5]. The results imply that the type I error does not change significantly as *N* varies.

**Table 1:** **Results of the simulation studies**.

		***V*_1_**	***V*_2_**	***N***	**min**	**max**	**σ**		**Type I error**
*H*_0_	Variation in the distance	40	200	12	0	1	1		0.051
		40	200	12	0.5	1.5	1		0S.053
		40	200	12	1.5	2.5	1		0.051
		40	200	12	3	5	1		0.059
		40	200	12	10	15	1		0.053
	Changing the percentage of inactivated voxels	20	220	12	0.5	1.5	1		0.069
		40	200	12	0.5	1.5	1		0.052
		80	160	12	0.5	1.5	1		0.062
		120	120	12	0.5	1.5	1		0.060
		200	40	12	0.5	1.5	1		0.056
	Varying the number of subjects	40	200	4	0.5	1.5	1		0.048
		40	200	8	0.5	1.5	1		0.052
		40	200	12	0.5	1.5	1		0.051
		40	200	20	0.5	1.5	1		0.058
		40	200	100	0.5	1.5	1		0.052
		***V*_1_**	***V*_2_**	***N***	**min**	**max**	σ	**σ_*b*_**	**Power**
*H*_*a*_	Basic alternatives—correlated	40	200	12	0.5	1.5	1	0.05	0.042
		40	200	12	0.5	1.5	1	0.1	0.059
		40	200	12	0.5	1.5	1	0.2	0.184
		40	200	12	0.5	1.5	1	0.5	0.972
		40	200	12	0.5	1.5	1	1	1.000
		40	200	12	0	1	1	0.2	0.195
		40	200	12	0.5	1.5	1	0.2	0.186
		40	200	12	1.5	2.5	1	0.2	0.196
		40	200	12	3	5	1	0.2	0.188
		40	200	12	10	15	1	0.2	0.195
		40	200	4	0.5	1.5	1	0.2	0.046
		40	200	8	0.5	1.5	1	0.2	0.101
		40	200	12	0.5	1.5	1	0.2	0.171
		40	200	20	0.5	1.5	1	0.2	0.342
		40	200	100	0.5	1.5	1	0.2	0.998
	Basic alternatives—uncorrelated	40	200	12	1	1	1	0.05	0.045
		40	200	12	1	1	1	0.1	0.065
		40	200	12	1	1	1	0.2	0.171
		40	200	12	1	1	1	0.5	0.973
		40	200	12	1	1	1	1	1.000
		***V*_1_**	***V*_2_**	***N***	**min**	**max**	σ	**σ_*a*_**	**Power**
	Variability of the angle of the principal axis—without angle correction	40	200	12	0.5	1.5	0.5	0.01	0.041
		40	200	12	0.5	1.5	0.5	0.02	0.051
		40	200	12	0.5	1.5	0.5	0.05	0.056
		40	200	12	0.5	1.5	0.5	0.1	0.025
		40	200	12	0.5	1.5	0.5	0.5	0.007
		40	200	12	0	1	0.5	0.01	0.042
		40	200	12	0.5	1.5	0.5	0.01	0.045
		40	200	12	1.5	2.5	0.5	0.01	0.054
		40	200	12	3	5	0.5	0.01	0.051
		40	200	12	10	15	0.5	0.01	0.030
	Variability of the angle of the principal axis—with angle correction	40	200	12	0	1	0.5	0.01	0.005
		40	200	12	0.5	1.5	0.5	0.01	0.027
		40	200	12	1.5	2.5	0.5	0.01	0.064
		40	200	12	3	5	0.5	0.01	0.078
		40	200	12	10	15	0.5	0.01	0.109
		***V*_1_**	***V*_2_**	***N***	**min**	**max**	σ	***V*_*a*_**	**Power**
	Changing activation sets	40	200	12	0.5	1.5	0.5	10	0.057
		40	200	12	0.5	1.5	0.5	20	0.048
		40	200	12	0.5	1.5	0.5	40	0.057
		40	200	12	0.5	1.5	0.5	100	0.097
		40	200	12	0.5	1.5	0.5	400	0.205

*Shown are type I error rates and power across simulation settings*.

#### 3.2.2. Simulation under the alternative hypothesis

There are a variety of ways in which the null hypothesis can fail to be true; herein, several key departures were analyzed. First, consider a straightforward departure, where Figure 1 holds, with sets (A) and (C) both empty. The extent of spherical and elliptical variation around the principal axis are evaluated. However, other departures could also be present. Most importantly, the null could be true for each subject, but with a varying angle along the principal axis. In addition, a non-trivial percentage of voxels changing activation status (i.e., sets (A) and (C) from Figure 1 being non-empty) would similarly represent a departure from the null hypothesis. The simulation scenarios for these parameters are described below.

The number of subjects remains *N* = 12 while *min* = 0.5, *max* = 1.5, *V*_1_ = 40, and *V*_2_ = 200.

**Figure 6:**
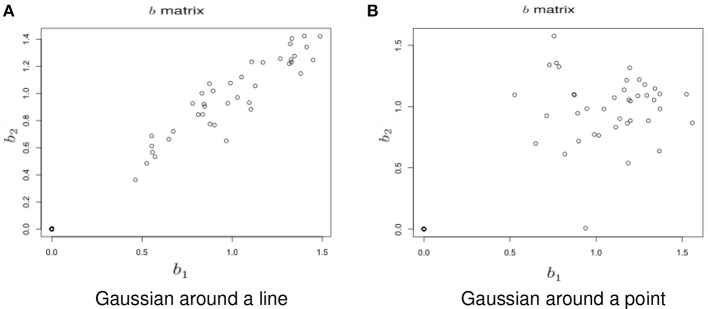
**Example simulation from the alternative hypothesis**. The axes are the two dimensional bivariate simulated data representing inter-session differences for each task in the motivating study. In **(A)** the voxels have Gaussian variation added orthogonally to the major axis. In **(B)** there is no relationship.

**Figure 7:**
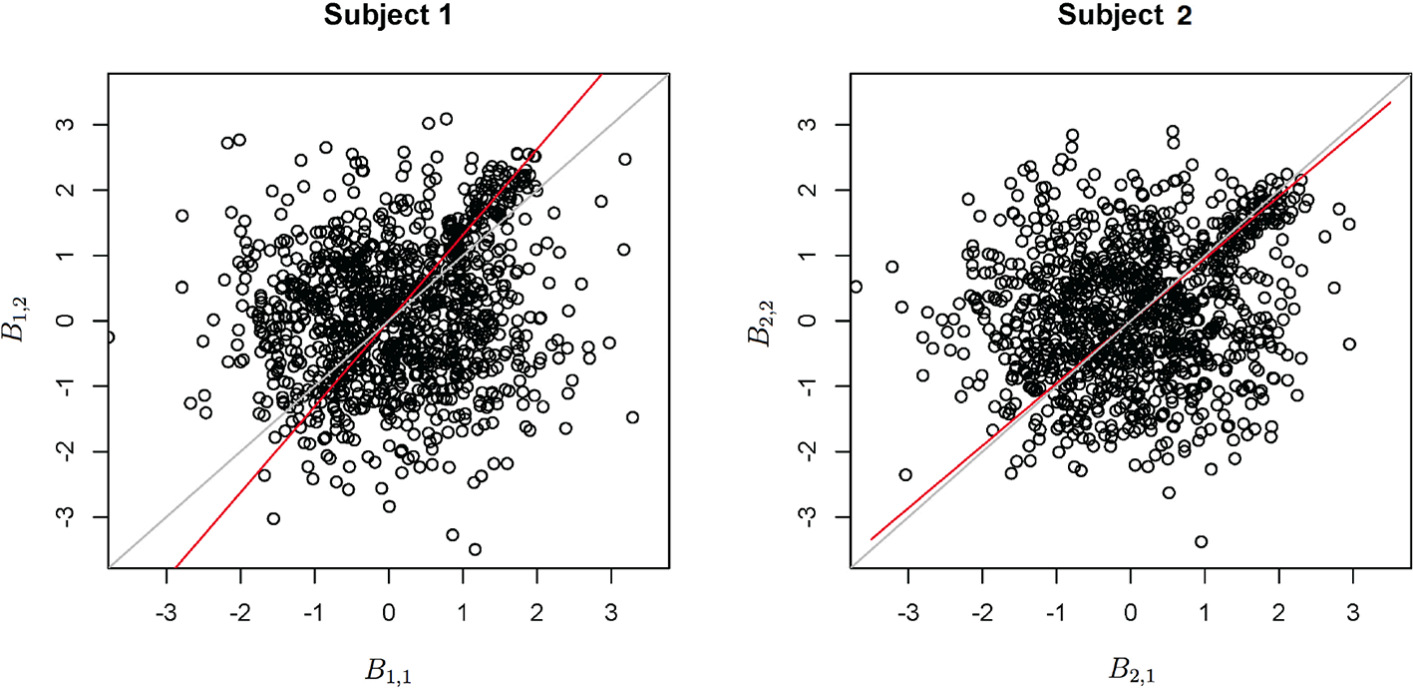
**Example simulation for the setting when the principal axis differs across subjects**. The axes are the two dimensional bivariate simulated data representing inter-session differences for each task in the motivating study. The gray line is a reference identity line, while the red line is the axis of principal direction.

**Figure 8:**
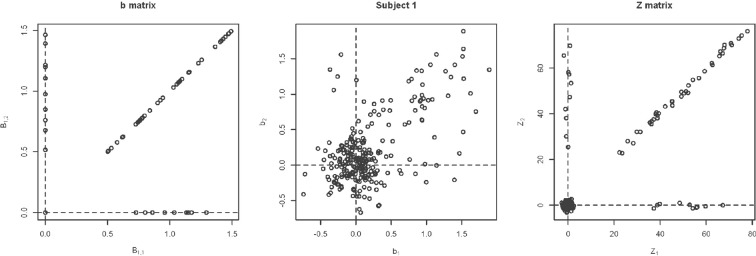
**Example simulation from the alternative with changing activation sets**. The axes are the two dimensional bivariate simulated data representing inter-session differences for each task in the motor learning study. Shown are the true parameter values (leftmost panel), the simulated subject data (middle panel) and the *Z*-values (rightmost panel).

**Simulation under a basic alternatives:** Two basic alternative settings were considered. In the first, the δ(*v*) were simulated as two dimensional, yet one dimension dominates the other. This method of simulation added orthogonal variation around the line used in the simulation under the null hypothesis. Specifically, the activated voxels have Gaussian variation orthogonal to the major axis (see Figure 6A). This was done in lieu of simulating a bivariate Gaussian with a non-zero correlation to consider an even, non-concentrated spread along the major axis. Simulations using a bivariate normal yielded similar results. In the second setting the correlation was assumed to be zero (see Figure 6B).
**Variability of the angle of the principal axis** Consider a null setting, as in Section 3.2.1. However, assume that the constant, *c*, varies across subjects. Let *c*_*i*_ denote this constant for subject *i*. To simulate the data, first the null simulation from Section 3.2.1 was performed then the observed bivariate points {*b*_1*iv*_, *b*_2*iv*_} were multiplied by the rotation $\text{matrix}\;(\begin{array}{cc} \cos\theta_{i} & -\sin\theta_{i}\\ \sin\theta_{i} & \cos\theta_{i} \end{array}),$ where θ_*i*_ is a subject-specific rotation angle from the 45^*o*^ line, generated from a Gaussian distribution with mean 0 and standard deviation σ_*a*_, which varied from 0.01 to 0.5. Before the rotation, *c* = 1, while afterwards, *c*_*i*_ = tan (45^*o*^ − θ_*i*_). Examples of the simulated data are shown in Figure 7.
**Changing Activation Sets** In this setting, the impact of a non-trivial percentage of voxels, or change in voxels that switch activation status, i.e., corresponding to a large collection of voxels in sets (A) and (C) in Figure 1. An example simulation is shown in Figure 8. Here, the *b*_*kiv*_ were either 0 or uniform, where a *min* = 0.5 and *max* = 1.5. The specific values were: 200 voxels set to be inactive for both the trained and untrained tasks, 40 voxels set to be activated for the trained and untrained groups, *V*_*a*_ voxels were activated with training, but inactivated without training, while another *V*_*a*_ voxels were inactivated with training but activated without training. Here *V*_*a*_ was varied between 10 and 400. Note that, in this setting, the *Z* matrix (see Figure 8) is substantially different from the direction of its first eigenvector.

### 3.3. Simulation results

Table 1 displays the results across the simulation settings. All tests were performed at a nominal 5% error rate.

#### 3.3.1. Simulations under the null hypothesis

Adherence to the specified nominal error rate was remarkably consistent as parameter settings varied. When varying the distance, the test showed only slight liberalism (Type I error rate larger than the nominal) across settings. Only for unrealistically small activation sets did the test demonstrate liberalism when altering the activation set size. In addition, varying the number of subjects had little impact. Adherence to the nominal error rate was acceptable, even at very low numbers of subjects.

#### 3.3.2. Simulations under the alternative hypothesis

Under the basic alternative, where the true voxel states possessed a strong (but not perfectly linear) corelation, power varied as expected. Under a strong correlation (σ_*b*_ close to 0), power trended to the nominal type I error rate. Encouragingly, power quickly trended to one as the true relationship moved away from a dominant dimension. As expected, the power tended to 1 as the sample size increased (confirming the relevant asymptotics). However, the sample size needed to be relatively large to have adequate power at the modest value of σ_*b*_ = 0.2.

In the case where no dimension dominated under the basic alternative of absence of correlation, power changed significantly with the spread of activation, σ_*b*_. When the angle of principal direction varied, power suffered dramatically. To address this, a first stage subject-specific principal components rotation was investigated. This appeared to improve power in settings where the null and non-null voxels were more clearly delineated, but continued to exhibit low power (11%) when the distance was large (min = 10, max = 15). A non-trivial fraction of voxels changing activation status had a negative impact on power.

## 4. Data analysis of the motivating data set

This section investigates the impact of training on activation using the APT data described in Section 3.1 and represented in Figures 3, 4, which show estimated beta maps. A null hypotheses suggests that the data points are close to the principal line. Notably, a distinction between the null and alternative hypothesis is difficult to ascertain graphically. However, it is apparent that the axis of principal direction varies by subject. Next, dimensionality is tested via three methods: first considering only the (trained) horizontal task, then only the (untrained) vertical task, and then comparing both. When considering the untrained task in isolation we are testing *H*_0_: β_21_(*v*) = *c*β_11_(*v*), then *H*_0_:β_22_(*v*) = *c*β_12_(*v*) for the trained and *H*_0_: β_21_(*v*) − β_11_(*v*) = *c*{β_22_(*v*) − β_12_(*v*)} when comparing trained and untrained. (The paper used the latter as the primary motivating example.) In Table 2, the results before and after angle correction are shown.

**Table 2:** ***P*-values of the tests of dimensionality for the motor learning data set**.

**Tasks**	**Without angle correction**	**With angle correction**
Horizontal Session 1 vs. Session 2	0.520	0.163
Vertical Session 1 vs. Session 2	0.598	0.050
Horizontal vs. Vertical		0.3620

*The first row considers the Session 1 vs. Session 2 for the Horizontal task (*H*_0_ : β_21_(*v*) = *c* β_11_(*v*)). The second row does the same for the vertical task (*H*_0_ : β_22_(*v*) = *c*β_12_(*v*)). The third considers inter-session differences across tasks (*H*_0_ : β_21_(*v*) - β_11_(*v*) = *c* {β_22_(*v*) - β_12_(*v*)}). P-values are given with and without having performed an angle correction*.

### 4.1. Motor learning data results

The axis of principal direction varied by subject (see Figures 3, 4). Before correcting for the principal angle, the tests of dimensionality were insignificant, for both the horizontal and the vertical tasks. However, after correcting the principal angle by subject, the *p*-values of the tests were highly reduced. Focusing only on the tasks separately, the test of dimensionality yielded a *p*-value of 0.05 for the vertical task and 0.16 for the horizontal one. When comparing across tasks, the *p*-value was 0.36. Thus, the untrained task has a significant second dimension that does not appear to be present in the trained. Inspecting the data, excess variability in the trained task appears to be due to biomodal changes in activation. It is not surprising that the comparison across tasks was non-significant, given the increased variability obtained from taking differences and the issues of power for the test.

## 5. Discussion

### 5.1. Simulation results

The simulation results suggest that tests of dimensionality are a reasonable exploratory testing procedure for investigating the distribution of paired activation maps. However, their confirmatory performance was hindered by instances with low power in situations that could be realistically seen in practice. The adherence to the nominal type I error rate, on the other hand, was uniformly acceptable across simulation settings. Thus, a rejection from this test is likely informative, while an acceptance less so.

The low power cases occurred where there is substantial variability in the principal axis, or where activation status changed. This latter condition created confusion between noise and signal, with the test attributing signal variability as noise. Of the two cases, careful masking could eliminate concern over changing activation status. However, variability in the principal axis is likely the norm and could arise from a number of plausible biological, technological and processing causes. The straightforward refinement of a first stage subject-level principal component rotation improves the power.

### 5.2. General discussion

This manuscript posited a different paradigm for statistically evaluating learning using task-related BOLD fMRI activation maps. At its core, the primary advance is the supposition of using the bivariate distribution of the activation maps, or changes in activation maps, when comparing tasks over sessions. Under this framework, changes in the *distribution* of activated voxels are key, not voxel level changes in activation extent, as would be evaluated in voxel-level parametric mapping interaction tests. An unintended benefit of this distributional approach in this setting is avoiding the familiar issue of having to determine interactions where main effects are not present.

**Figure 9:**
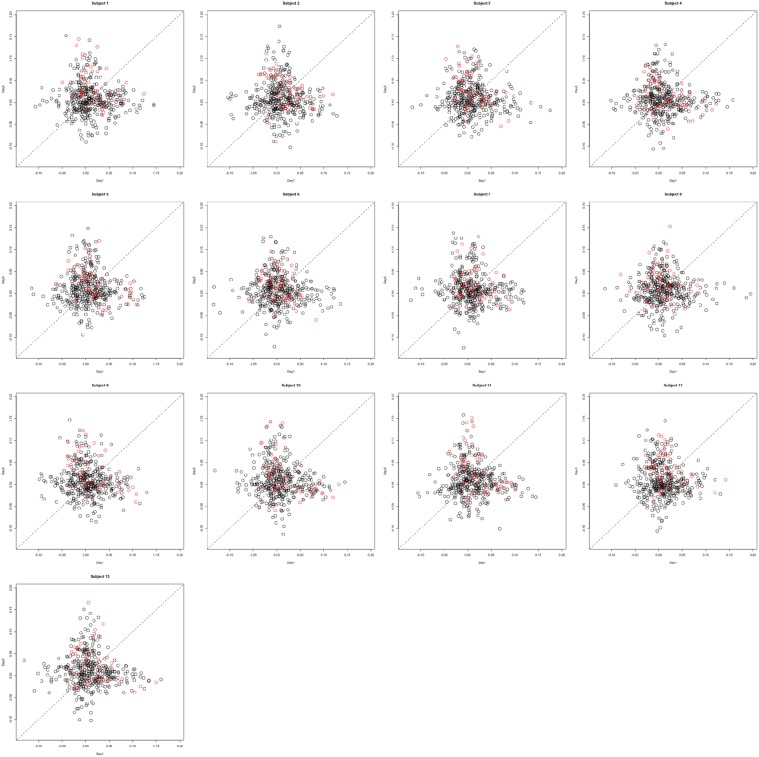
**A simulation example highlighting increased power for detecting learning based differences**. The axes are the two dimensional bivariate simulated data representing inter-session differences for each task in the motivating study. The alternative of the dimensionality test is true and the *P*-value is 0.03, suggesting that activation extent is unrelated between tasks. However, only 11% of the voxels satisfy a voxel level test of significance (colored in red).

The intended benefit of increasing power over voxel-level interaction tests was found to be true, provided assumptions hold. For example, Figure 9 provides a simulation example where the alternative test of dimensionality is both true and detected (*P*-value of 0.03). However, only 11% of the voxels would satisfy a voxel level test of significance. We emphasize the different nature of the hypotheses interrogated by these approaches so that comparisons of power should be taken with a grain of salt.

Evaluating distributional differences for learning-based activation tests a different scientific hypothesis than voxel level testing. In our example, the question was how BOLD activation, or changes in activation, relate between trained and untrained tasks. Investigating activation distributions is less sensitive to the requirement of focal localization of effects compared to interaction testing. For example, two small spatially separated significant interaction regions may have different voxel-level interaction significance than a single contiguous region of the same aggregate size. In contrast, the distribution may not change. Conversely, evaluating contrast map distributions does not provide the benefits of localization to inform results.

It is worth emphasizing that the investigation of activation distribution represents a complementary procedure to voxel-level testing and does not represent a form of omnibus test to be performed prior to it. Thus, it is perhaps not useful to generate a single analytic pipeline, whereby omnibus distributional tests are followed by voxel level contrasts of interest.

An interesting next direction in this line of research would consider full models of the joint distribution of {β_11_(*v*), β_12_(*v*), β_21_(*v*), β_22_(*v*)}. This could be accomplished using a Bayesian random effects approach via mixtures of Gaussian random variables. However, the feasibility, applicability and gain of such an approach over simpler solutions remains unknown. A tantalizing possible benefit would be robustness to inter-subject registration to a template. In contrast, interaction tests focus on localization and as such, place a heavy burden on accurate inter-subject registration. A full random effect mixture model could possibly remove the need for inter-subject registration, or at least remove the need for non-affine registration.

The far simpler approach discussed in this manuscript addresses dimensionality. The results show that the operating characteristics of the approach are viable, if modeling assumptions are met. Particularly encouraging was the robustness to variation in the distance of the center of activation from null voxels. However, its sensitivity to the angle of the principal axis is a core issue, as such variation is clear from the data.

In the real data analysis it is noteworthy that the vertical and horizontal tasks differed in their respective tests of dimensionality. Particularly, the null hypothesis was not rejected in the trained task (horizontal) while it was in the untrained task (vertical). However, there does appear to be more apparent non-Gaussianity in the vertical task, suggesting a component of the rejection is related to a form of dimensionality not well-covered by the model. The contrast test comparing vertical vs. horizontal was not significant. Therefore, it cannot be concluded that the activation distribution given by the inter-session differences across tasks is not linear. For all three cases, the data analysis suggests large variability in the subject-specific principal axes, a setting where low power was evidenced in the simulation study. Thus, the null results are perhaps indicative of low power.

### Conflict of interest statement

The authors declare that the research was conducted in the absence of any commercial or financial relationships that could be construed as a potential conflict of interest.
